# Epigenetic silencing of *miR-137* contributes to early colorectal carcinogenesis by impaired *Aurora-A* inhibition

**DOI:** 10.18632/oncotarget.12719

**Published:** 2016-10-18

**Authors:** Yu-Chuan Huang, Chung-Ta Lee, Jenq-Chang Lee, Yao-Wen Liu, Ying-Jen Chen, Joseph T. Tseng, Jui-Wen Kang, Bor-Shyang Sheu, Bo-Wen Lin, Liang-Yi Hung

**Affiliations:** ^1^ Institute of Bioinformatics and Biosignal Transduction, National Cheng-Kung University, Tainan 70101, Taiwan; ^2^ Department of Biotechnology and Bioindustry Sciences, National Cheng-Kung University, Tainan 70101, Taiwan; ^3^ Department of Life Sciences, College of Bioscience and Biotechnology, National Cheng-Kung University, Tainan 70101, Taiwan; ^4^ Center for Infectious Disease and Signal Transduction Research, College of Medicine, National Cheng-Kung University, Tainan 70101, Taiwan; ^5^ Department of Pathology, National Cheng-Kung University Hospital, Tainan 70403, Taiwan; ^6^ Department of Internal Medicine, National Cheng-Kung University Hospital, Tainan 70403, Taiwan; ^7^ Department of Surgery, National Cheng-Kung University Hospital, Tainan 70403, Taiwan; ^8^ Department of Pathology, Kuo General Hospital, Tainan 70054, Taiwan; ^9^ Institute for Cancer Biology and Drug Discovery, College of Medical Science and Technology, Taipei Medical University, Taipei 11031, Taiwan

**Keywords:** miR-137, Aurora-A, COX2, colorectal cancer, epigenetic regulation

## Abstract

*MicorRNA-137* is silenced in human colorectal cancer tissues and colon polyps. Our study showed that the decreased expression of *miR-137* is significantly different in various types of polyp which maintain different potentials to lead to CRC development. The expression of *miR-137* gradually decreases during the process of colorectal carcinogenesis. Receiver operating characteristic curve (ROC) analysis indicates that the loss of *miR-137* expression in colon polyps can serve as a biomarker to predict the predisposition of colorectal carcinogenesis. By cell model and xenograft animal model, the enforced expression of *miR-137* in colorectal cancer cells can inhibit cell proliferation and tumor formation, induce G2/M arrest, and lead to apoptosis. The expression pattern of *miR-137* and *Aurora-A* or *PTGS2* is negatively correlated in human colorectal cancer tissues and colon polyps. Those effects induced by overexpressed *miR-137* can be rescued by the overexpression of Aurora-A. In summary, our study suggests that the loss of *miR-137* expression in colon polyps can serve as a biomarker to predict the tendency toward to CRC formation through the impaired inhibitory effect of Aurora-A. The investigation of the regulatory mechanism of *miR-137*-mediated Aurora-A inhibition may shed new light on the early prognosis of cancer therapy for CRC in the future.

## INTRODUCTION

Colorectal cancer (CRC) is the third most commonly diagnosed cancer, and the second leading cause of cancer-related deaths worldwide. The five-year survival rate for early-stage CRC is approximately 90%, whereas it decreases to only 12% for patients diagnosed with late-stage CRC. The development of CRC progresses consecutively in the adenoma-carcinoma pathway, which is a neoplastic pathway involved in the formation of various solid tumors. In addition to the neoplastic pathway, the chromosomal instability (CIN) pathway, microsatellite instability (MSI) pathway, CpG island methylation pathway (CIMP), and serrated neoplasia pathway all have been reported to participate in the formation of CRC individually or concurrently [1–3]. According to clinical observations, only a small population of polyps can develop into CRC; and the formation of conventional adenoma remains the only well-recognized precursor of CRC. The recurrence rate following surgery is 10% in stage I patients, 30–40% in stage II patients, and 60–70% in stage III patients. Therefore, early diagnosis and therapy in colorectal cancer patients has a significant prognostic value, and it is an urgent necessity to identify new biomarkers or specific therapeutic targets for CRC.

Aurora-A is aberrantly overexpressed in various cancer types, including CRC, and plays an important role in the tumorigenesis [4]. Overexpression of Aurora-A disturbs the cell cycle checkpoint, impairs centrosome function and chromosome segregation, induces CIN, and promotes the epithelial-mesenchymal transition (EMT) [5]. All of these effects of overexpressed Aurora-A make it a good target for cancer therapy. Currently, there are several Aurora kinase inhibitors under clinical trials in patients with various solid tumor malignancies and hematologic cancers [4]. Prostaglandin-endoperoxide synthase 2 (PTGS2), also known as cyclooxygenase 2 (COX2), plays a critical role in colorectal carcinogenesis [6, 7]. The expression of PTGS2 is elevated in CRC tissues. The *PTGS2* transgenic mouse has a higher risk than the wild-type mouse to induce the formation of colorectal cancer tumors after treatment with AOM (azoxymethane) [8]. It is believed that the regular use of PTGS2 inhibitors, such as aspirin, can reduce the risk of colorectal cancer [9].

The expression of microRNAs (miRNAs) can be used as biomarkers in the early diagnosis or prognosis of cancers [10]. According to the literatures, miRNAs can be overexpressed or repressed in CRC, and mechanically act as oncogenes or tumor suppressor genes [11, 12]. *MiR-137*, a tumor suppressor gene, is epigenetically silenced in colorectal cancer [13–15]. Several reports indicated that *miR-137* negatively regulates the progression of CRC through directly targeting the oncogenes, such as Musashi-1, paxillin, FMNL2 and Cdc42 [16–19]. In addition, *miR-137* may cooperate with other miRNAs to inhibit the growth of CRC [20]. In this report, we identified *miR-137* as a potential biomarker to predict the risk of colorectal carcinogenesis. The expression of *miR-137* is differentially reduced in different types of colon polyps, the early-stage of pre-cancerous lesions of CRC, with different potencies to CRC development. The early loss of *miR-137* has a higher risk of colorectal carcinogenesis. During colorectal carcinogenesis, *miR-137* is silenced through epigenetic regulation. The enforced expression of *miR-137* in CRC can repress the cell proliferation and induces cell apoptosis. Therefore, in addition to its role as a biomarker, *miR-137* may serve as a therapeutic miRNA in CRC.

## RESULTS

### Epigenetic silencing results in the loss of *miR-137* expression in colorectal cancer cells and polyps

**Figure 1:**
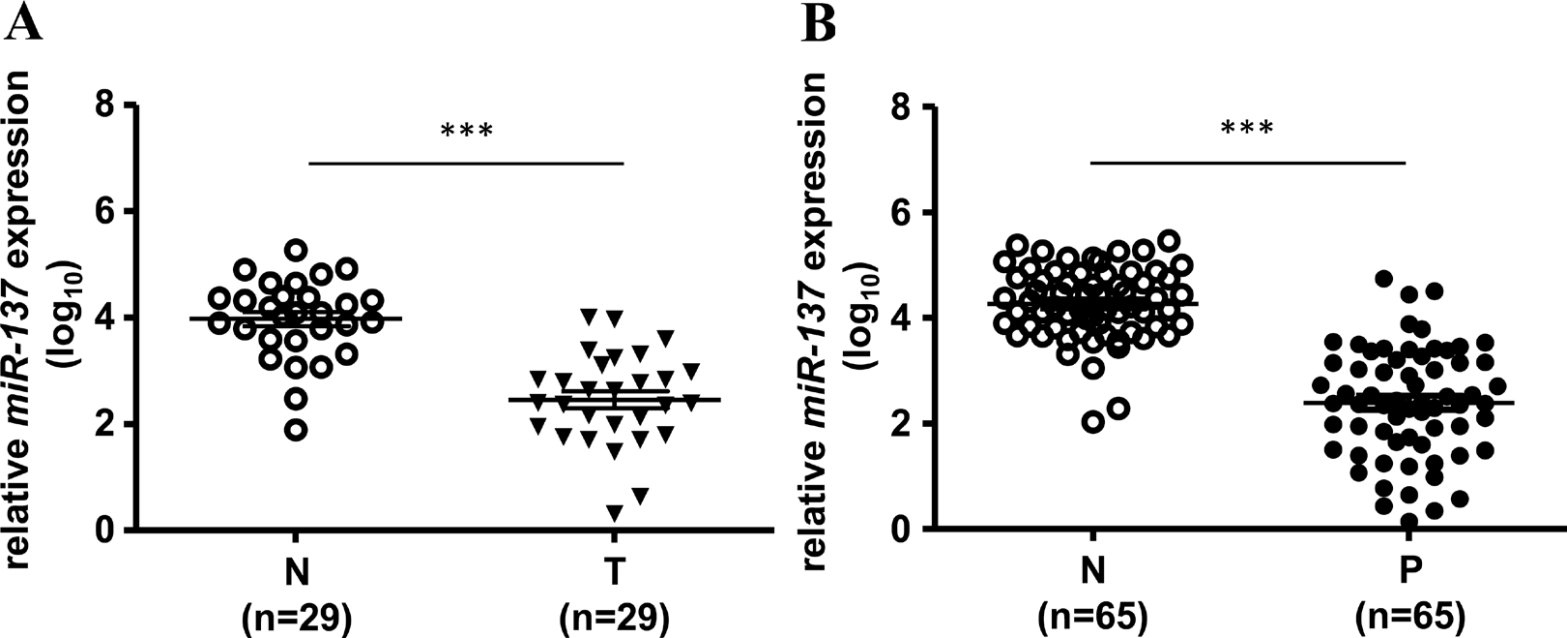
The expression of *miR-137* is decreased in human colorectal cancer tissues and colon polyps The expression level of *miR-137* in 29 paired colorectal cancer tissues (T) and adjacent normal tissues (N) (**A**) or 65 paired colon polyps (P) and adjacent normal tissues (N) (**B**) was analyzed by TaqMan Q-PCR. ***p < 0.001 by Mann-Whitney *U-test*. Bars represent standard deviation of the mean (SEM).

We first checked the expression status of *miR-137* in human colorectal cancer cell lines and colorectal cancer tissues. The results showed that the expression of *miR-137* was almost undetectable in all of the tested colorectal cancer cell lines (Supplementary Figure S1A) and decreased in human colorectal cancer (CRC) tissues compared with the paired adjacent normal mucosa (Figure 1A and Supplementary Table S1). The decreased level of *miR-137* showed no difference between the early stage and late stage of CRC (Supplementary Figure S1B). Interestingly, we found that the expression of *miR-137* was also decreased in colon polyps, the pre-cancerous lesions of CRC (Figure 1B and Supplementary Table S2). The *miR-137* expression level was gradually decreased from normal colon mucosa, polyps to colorectal cancer tissues (Supplementary Figure S1C). These results imply that the loss of *miR-137* expression may occur in the early carcinogenesis of colorectal cancer.

**Figure 2:**
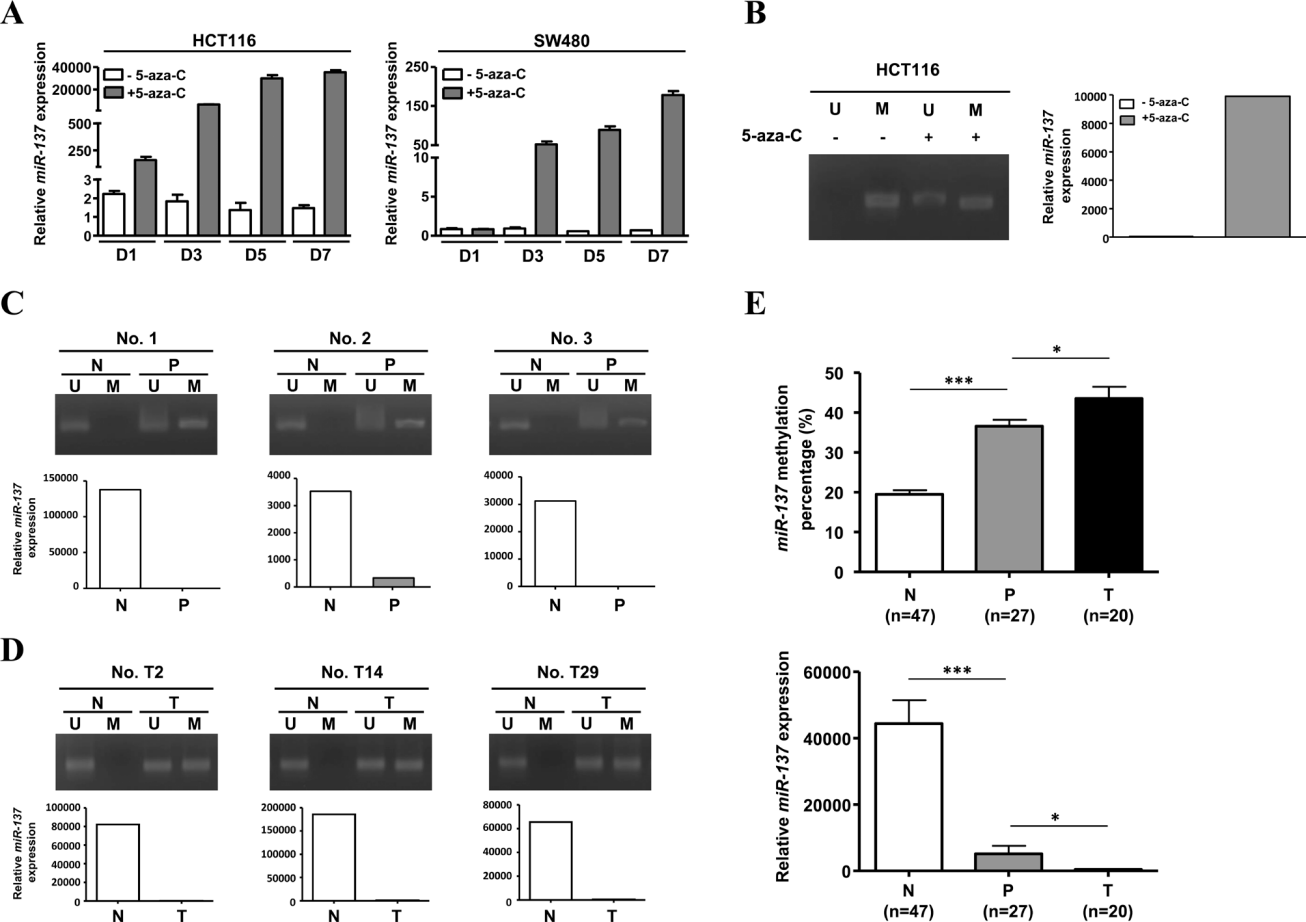
*miR-137* is methylated in colorectal cancer cells (**A**) HCT116 and SW480 cells treated with (+) or without (−) 2.5 μM 5-aza-CdR for 1, 3, 5, and 7 days (D1, D3, D5 and D7) were harvested to determine the expression of *miR-137*. (**B**) HCT116 cells treated with (+) or without (−) 5-aza-CdR for 7 days were collected to analyze the methylation status and expression level of *miR-137* by MSP and Q-PCR, respectively. (**C**–**D**) The methylation status and expression level of *miR-137* in colon polyps (P) (C), colorectal cancer (T) (D) and their adjacent normal tissues (N) were analyzed as described above. Six representative specimens of polyps (No. 1, No. 2 and No. 3) and colorectal cancer tissues (No. T2, No. T14 and No. T29) are shown. (**E**) Twenty-seven human colon polyps (P), twenty human colorectal cancer tissues (T) and their adjacent normal tissues (N) were collected to check the methylated level (upper) and expression level (lower) of *miR-137* by pyrosequencing analysis and Q-PCR, respectively. The quantitative results of the methylated *miR-137* are shown as percentages (%). ***p < 0.001 and *p < 0.05 by Mann-Whitney *U-test*.

According to a previous report, the expression of *miR-137* can be epigenetically regulated [13]. We checked the *miR-137* genome and found that there are CpG islands spread throughout the promoter region and *miR-137* transcript (Supplementary Figure S2A). When colorectal cancer cells were treated with 5-aza-2′-deoxycytidine (5-aza-C), a methyl transferase inhibitor, the expression of *miR-137* was induced (Supplementary Figure S2B). The 5-aza-C-induced expression of *miR-137* in CRC cell lines is time dependent (Figure 2A). Methylation-specific PCR (MSP) further confirmed the methylation status of *miR-137* in the colorectal cancer cell line HCT116, human polyps and colorectal cancer tissues, whereas both 5-aza-C-treated cells and normal colon mucosa showed an un-methylated pattern of *miR-137* (Figure 2B–2D). The expression level of *miR-137* in HCT116, human polyps and colorectal cancer tissues was determined by Q-PCR accordingly (Figure 2B–2D). Furthermore, the methylation of *miR-137* in human normal mucosa and polyp was quantified by pyrosequencing (Supplementary Figure S3). The results showed that the methylation percentage of human colon mucosa is around 20%, and that of polyps and colorectal cancer tissues is around 40% to 50%, and the expression level of*miR-137* is negatively correlated with the methylation status (Figure 2E). These data suggest that *miR-137* is epigenetically regulated during colorectal cancer progression as previously shown [13, 14].

### *miR-137* targets several important genes that are involved in the tumorigenesis of colorectal cancer

**Figure 3:**
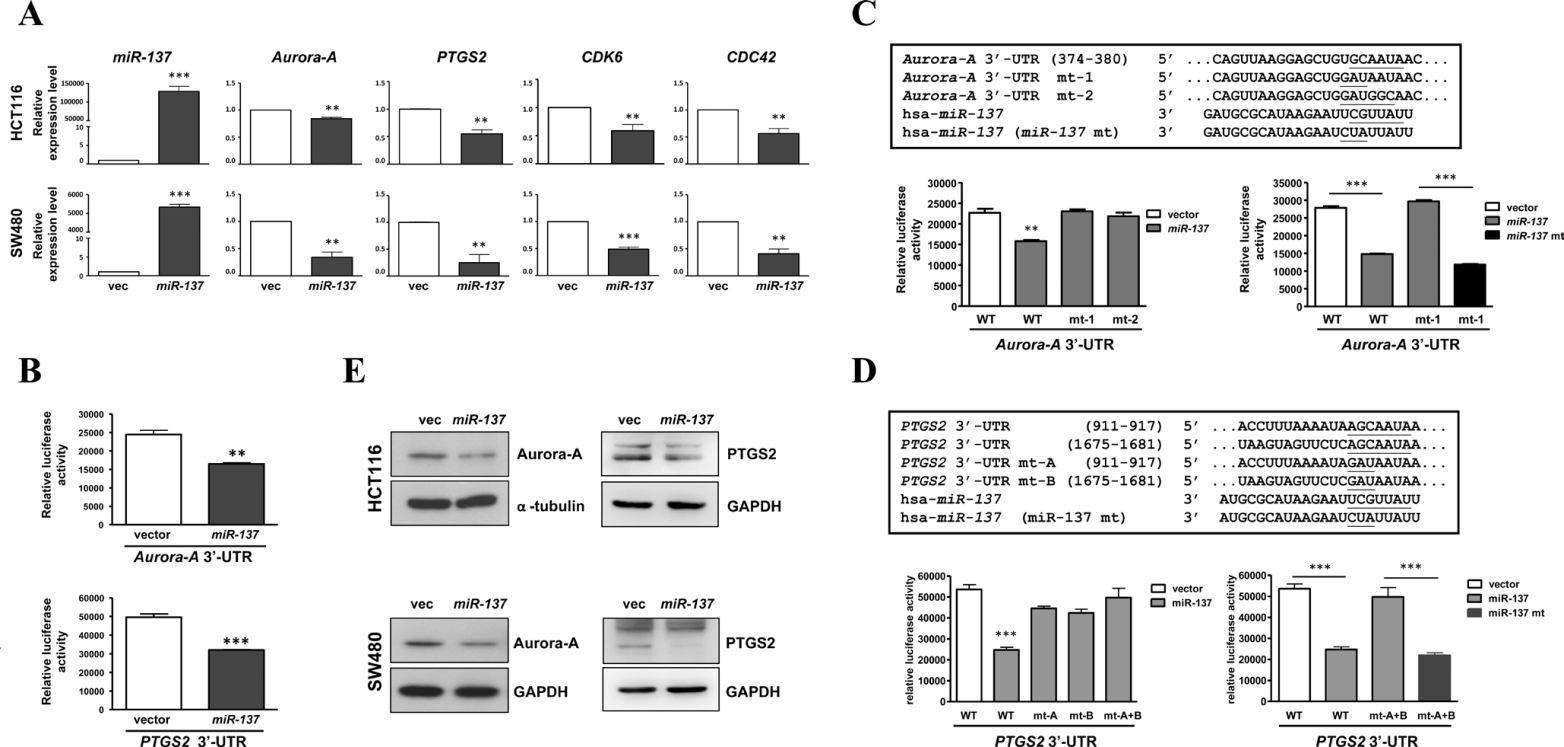
*mir-137* specifically targets *PTGS2*, *CDK6*, *CDC42* and *Aurora-A* (**A**) HCT116 and SW480 cells were transiently transfected with *miR-137* or vector control. The expression level of *miR-137* and its target genes *Aurora-A*, *PTGS2*, *CDK6*, and *CDC42* was determined by TaqMan Q-PCR. (**B**) HCT116 cells were co-transfected with *miR-137* or vector control and *Aurora-A* 3′-UTR (upper) or *PTGS2* 3′-UTR (lower), and then the luciferase reporter assay was performed. **(C–D)** A schematic diagram of *Aurora-A* 3′-UTR wild type (WT) and mutated sequences (mt-1 and mt-2) (C); and *PTGS2* 3′-UTR wild type (WT) and mutated sequences (mt-A and mt-B) (D) in the *miR-137* targeting site. The *miR-137* mutant (miR-137 mt) which complements the *Aurora-A* 3′-UTR mt-1 and two *PTGS2* 3′-UTR mutants, mt-A and mt-B, of *is* shown below. Cells co-transfected with *Aurora-A* or *PTGS2* 3′-UTR wild type or mutants and *miR-137* or *miR-137* mutant (miR-137 mt) were collected for reporter assays to check the specificity of *Aurora-A* or *PTGS2* 3′-UTR and *miR-137*. (**E**) HCT116 and SW480 cells transiently transfected with *miR-137* were collected to determine the expression levels of Aurora-A and PTGS2 by Western blot analysis.

To investigate the effects of *miR-137* in colorectal tumorigenesis, we searched its targeting genes through miRanda and TargetScan, confirmed by miRTarBase (Supplementary Figure S4A). MetaCore analysis showed that those potential *miR-137*-targeted genes are involved in pathways which are involved in cancer development or malignancies (Supplementary Figure S4B). Four known candidate genes, *Aurora-A*, *PTGS2*, *CDK6* and *CDC42*, were selected for further validation (Supplementary Figure S4C). By RT-qPCR, the expression of *Aurora-A*, *PTGS2*, *CDK6* and *CDC42* was decreased in *miR-137*-overexpressing HCT116 and SW480 cells (Figure 3A). The luciferase reporter assay using the 3′-UTR of *Aurora-A* or *PTGS2* mRNAs indicated that the overexpression of *miR-137* can down-regulate *Aurora-A* and *PTGS2* (Figure 3B).

We further checked the specificity of *miR-137* in targeting *Aurora-A* and *PTGS2* using two mutants of *Aurora-A* 3′-UTR (Figure 3C, *Aurora-A* 3′-UTR mt-1 and *Aurora-A* 3′-UTR mt-2), two mutants of *PTGS2* 3′-UTR (Figure 3D, *PTGS2* 3′-UTR mt-A and *PTGS2* 3′-UTR mt-B), and a seed region mutant of *miR-137* that can complementarily target the *Aurora-A 3′-UTR* mt-1 and *PTGS2* 3′-UTR mt-A and mt-B (Figure 3C and 3D, *hsa-miR-137* mt). The luciferase reporter assay indicated that wild-type *miR-137* can only inhibit the wild-type *Aurora-A* 3′UTR and *PTGS2* 3′-UTR but not the mutants of *Aurora-A* 3′-UTR or *PTGS2* 3′-UTR, whereas the *miR-137* mt can specifically inhibit the *Aurora-A* 3′-UTR mt-1 (Figure 3C) and *PTGS2* 3′-UTR mt-A and mt-B (Figure 3D). Furthermore, the expression of Aurora-A and PTGS2 was also decreased in *miR-137*-overexpressing cancer cell lines (Figure 3E).

### Overexpression of *Aurora-A* and *PTGS2* occurs in colon polyps and has a reverse correlation with *miR-137* in both colon polyps and colorectal cancer tissue

**Figure 4:**
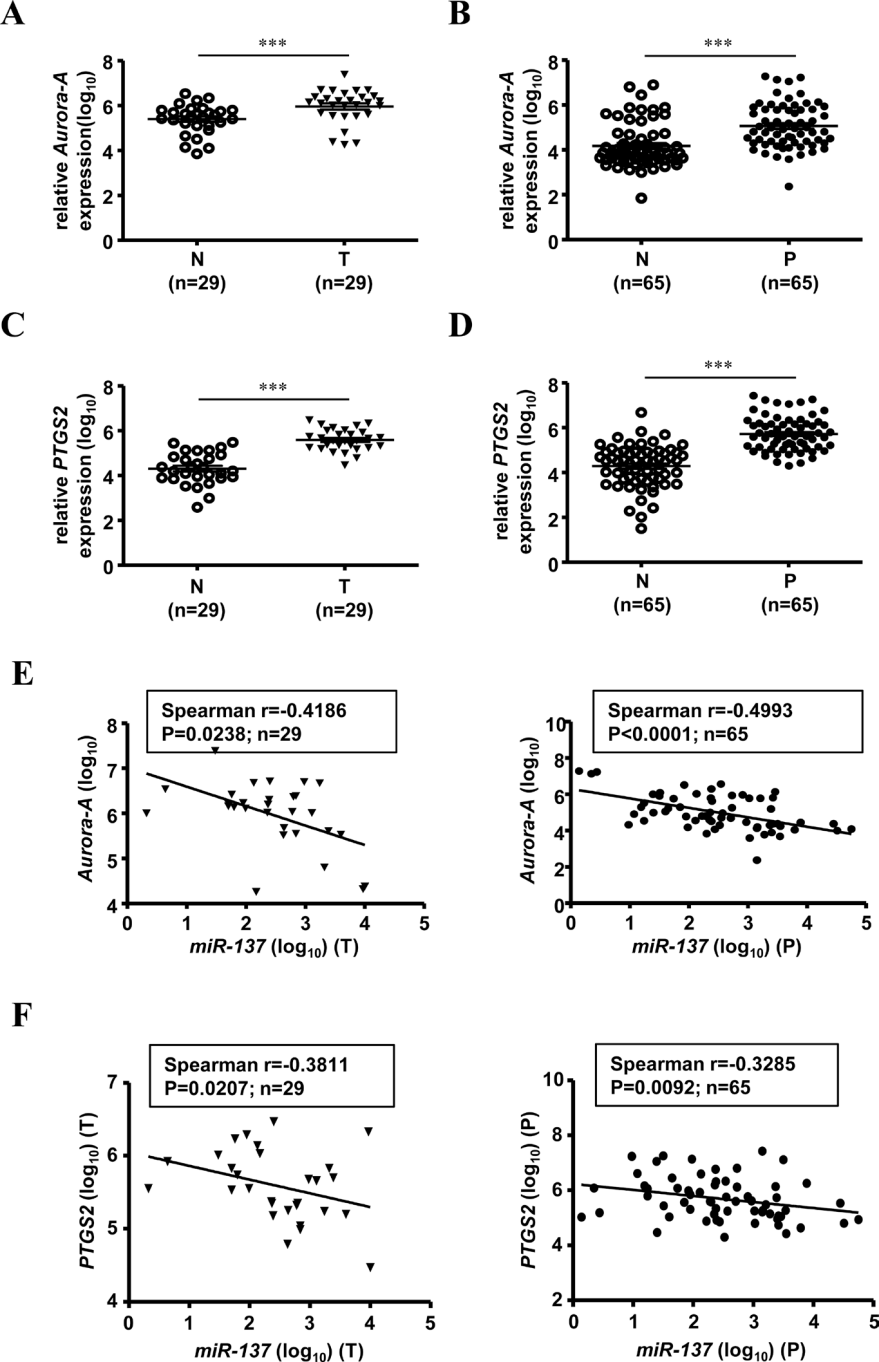
The expression of *miR-137* is negatively correlated with *Aurora-A* mRNA in human colorectal cancer tissues and colon polyps Total RNA purified from tumorous tissues of CRC (T) (**A**, *N* = 29) or colon polyps (P) (**B**, *N* = 65) was collected to determine the expression levels of *Aurora-A* mRNA by TaqMan Q-PCR. The expression level of *Aurora-A* mRNA is shown as log_10_. (**C**–**D**) The expression level of *PTGS2* mRNA was determined by Q-PCR as described above. (**E**–**F**) The negative correlation between *miR-137* and *Aurora-A* (E) or *PTGS2* (F) in patients with colorectal cancer (left) or colon polyps (right) is shown. The tumorous tissues of CRC (T) and colon polyps (P) are the same as those in Figure 1.

Our previous results showed that the expression of *miR-137* was decreased in human colorectal cancer tissues and colon polyps (Table 1 and Figure 1A–1B). Here, we checked the expression level of *Aurora-A* in clinical colorectal cancer tissues and colon polyps using the same cohort of patients. As expected, *Aurora-A* is overexpressed in the tumorous tissues of human colorectal cancer (Figure 4A). Surprisingly, the expression level of *Aurora-A* was increased in the colon polyps, the pre-cancerous lesions of CRC (Figure 4B). The same expression pattern was observed in *PTGS2*, which is an important gene involved in the development of CRC (Figure 4C and 4D) [21]. Interestingly, the increased expression levels of *Aurora-A* mRNA and *PTGS2* mRNA in different stages of CRC tissues were not statistically significant (Supplementary Figure S5A), whereas gradually increased *Aurora-A* mRNA expression was noted from normal mucosa, colon polyps to CRC tissues (Table 1 and Supplementary Figure S5B). Expression of Aurora-A was gradually increased in colon polyps and colorectal cancer; not surprisingly, the expression level of *PTGS2* mRNA is no different in colon polyps and colorectal tumorous tissues (Table 1 and Supplementary Figure S5B). The expression of *miR-137* and *Aurora-A* or *PTGS2* was negatively correlated in both human colorectal cancer tissues and colon polyps (Figure 4E–4F).

**Table 1:** Association between *miR-137*, *Aurora-A* and *PTGS2* expression with colorectal cancer (Tumor) and (polyp)

	Polyp (*n* = 64)	Tumor (*n* = 29)	*p*-value
**Age**			0.839
30–50 y/o (*n* = 9)	9 (9.5%)	3 (3.2%)	
51–80 y/o (*n* = 51)	56 (59.6%)	26 (27.7%)	
**Gender**			0.073
Male (*n* = 45)	48 (51.1%)	16 (17.0%)	
Female (*n* = 15)	17 (18.1%)	13 (13.8%)	
***miR-137*** **(mean log_10_ = 2.79)**			**0.009**
high (log_10_ > 2.79)	39 (41.5%)	9 (9.6%)	
low (log_10_ < 2.79)	26 (27.7%)	20 (21.3%)	
***Aurora-A*** **(mean log_10_ = 4.64)**			**< 0.0001**
high (log_10_ > 4.64)	23 (24.5%)	25 (26.6%)	
low (log_10_ < 4.64)	42 (44.7%)	4 (4.3%)	
***PTGS2*** **(mean log_10_ = 5.26)**			0.797
high (log_10_ > 5.26)	31 (33.0%)	13 (13.8%)	
low (log_10_ < 5.26)	34 (36.2%)	16 (17.0%)	

Bold values indicate statistically significant (Chi square).

### *miR-137* targets *Aurora-A* to arrest cell cycle progression and apoptosis

**Figure 5:**
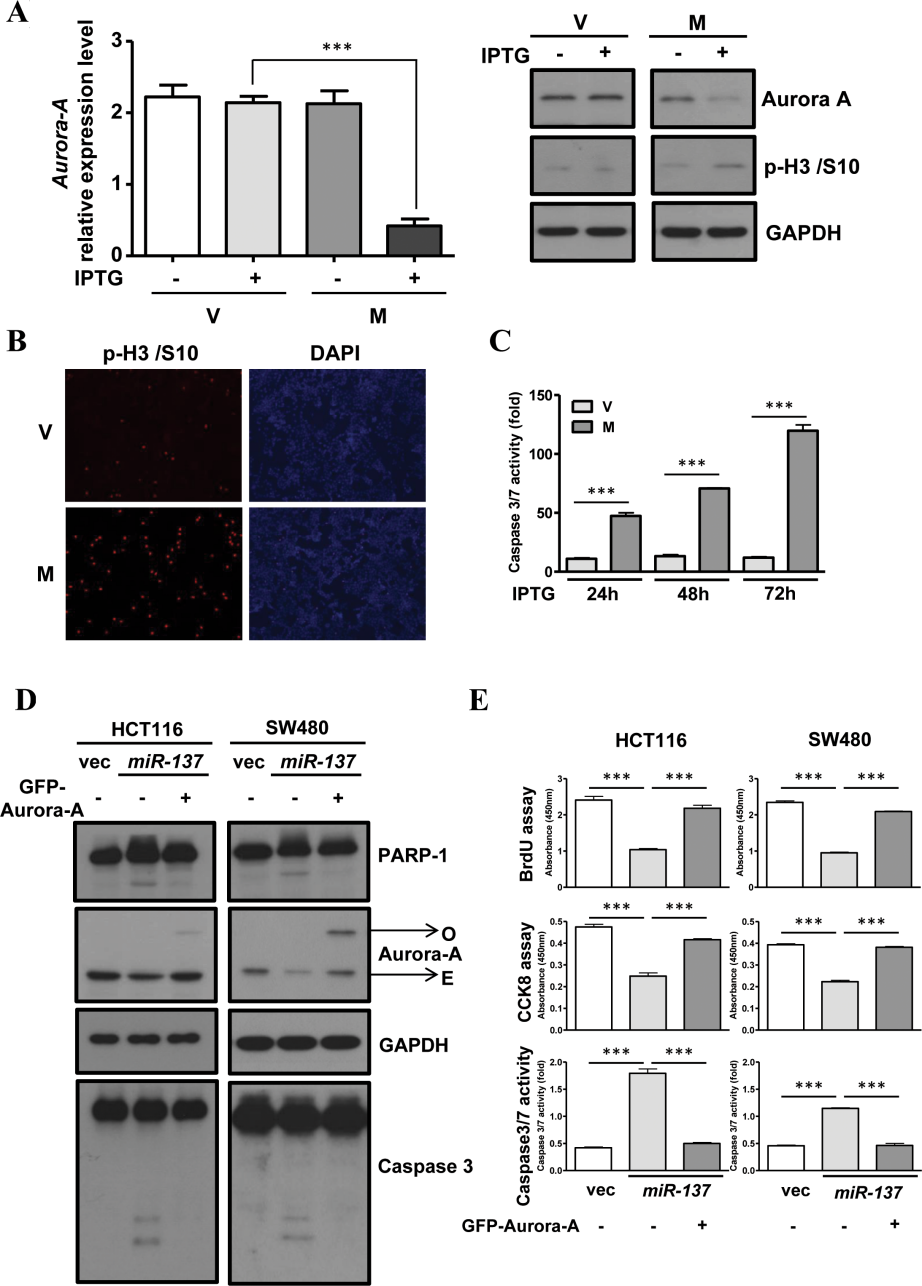
The overexpression of Aurora-A rescues the effect of *miR-137*-induced G2/M accumulation and apoptosis (**A**–**B**) Vector control (V) or *miR-137* (M) stably expressed HCT116 cells were treated with IPTG for 48 h to determine the expression level of *Aurora-A* mRNA (A, left) and protein (A, left). The expression level of phosphor-Histone H3/Ser10 (p-H3/S10) was determined by Western blot analysis (A, left) or immunofluorescence assay (B). (**C**) Caspase3/7 activity was determined in vector control (V) or *miR-137* (M) stably expressed HCT116 cells upon IPTG treatment for 24 h, 48 h and 72 h. (**D**–**E**) Vector control (V) or *miR-137* (M) stably expressed HCT116 and SW480 cells were transfected with (+) or without (−) GFP-Aurora-A. The cell proliferation ability was determined by the BrdU incorporation assay and CCK8, and apoptosis was detected by measuring the caspase3/7 activity (D). Total cell lysates were collected to perform Western blot analysis using antibodies as indicated (E).

Due to the facts that the other three genes, *PTGS2*, *CDK6* and *CDC42*, have been reported [15, 22–25], and the functional correlation between *miR-137* and *Aurora-A* in the early stage of colorectal carcinogenesis is still unclear, here, we focused on the investigation of the *miR-137*-*Aurora-A* axis in colorectal carcinogenesis. To evaluate the effect of *miR-137* in tumorigenesis, the IPTG-induced *miR-137* HCT116 stable expression cell line was established. After the addition of IPTG, the expression of *miR-137* was induced (Supplementary Figure S6) and Aurora-A expression was decreased (Figure 5A). The expression of *PTGS2*, *CDK6* and *CDC42* was also decreased in *miR-137*-expressing stable cells (Supplementary Figure S7). Western blot analysis and immunofluorescence assay showed that phosphorylated histone H3/S10, which is an indicator of mitotic cells and Aurora-A inhibition, is increased in IPTG-treated *miR-137* stable cells (Figure 5A and 5B) or *miR-137* transiently transfected cells (Supplementary Figures S8A and S8B). Flow cytometry showed that the overexpression of *miR-137* leads to an increase in the G2/M population of HCT116 or SW480 cells (Supplementary Figure S8C).

Given the effect of Aurora-A inhibition in inducing cell apoptosis, we analyzed the effect of *miR-137* expression in apoptosis. The results showed that IPTG treatment induced the activation of Caspase-3/7 in a time-dependent manner in stable cell lines (Figure 5C). The same effects were observed in *miR-137* transiently transfected HCT116 or SW480 cells. The cleavage of PARP and caspase-3 is increased in *miR-137*-expressing cells (Supplementary Figure S8D). The activities of caspase-3/7 and the sub-G1 population were augmented in *miR-137*-expressing cells in a time-dependent manner (Supplementary Figure S8E). Both the BrdU incorporation assay and cell proliferation assay indicated that the overexpression of *miR-137* can halt the cell proliferation rate (Figure 5E). Interestingly, ectopic expression of GFP-Aurora-A can rescue *miR-137*-induced proliferative inhibition and cell apoptosis (Figure 5D–5E).

Our previous results showed that *miR-137* is epigenetically silenced in cultured colorectal cancer cell lines, and treatment with 5-aza-C can induce its expression (Supplementary Figure S2 and Figure 2A). Consistently, when HCT116 cells were treated with 5-aza-C, the expression of Aurora-A was decreased and that of cleavage PARP and caspase-3 was increased, which are the phenomena similar to overexpressed *miR-137* (Supplementary Figure S9).

### Inducing the expression of *miR-137* impairs the tumor growth ability *in vivo*

**Figure 6:**
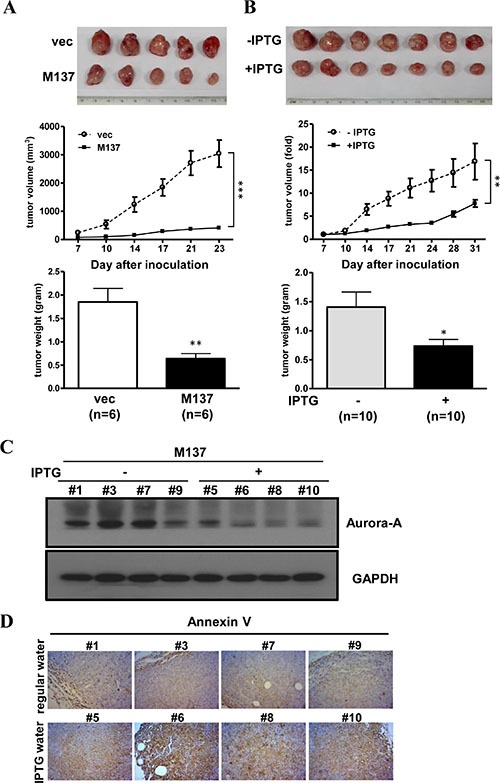
The stable expression of *miR-137* represses the tumor formation ability (**A**) A total of 1 × 10^6^ vector- or *miR-137*-stably expressing HCT116 cells was subcutaneously injected into NOD-SCID mice, and then the mice were supplied with IPTG water. The gross view (upper), tumor volume (middle) and tumor weight (lower) are shown. The tumor volume is calculated using the formula: length × width^2^ × 0.5. (**B**) A total of 1 × 10^6^ *miR-137*-stably expressing HCT116 cells were subcutaneously injected into NOD-SCID mice. After the tumors grew to approximately 100 mm^3^, the mice were divided randomly into two groups to supply with IPTG water (+) or regular water (−). The growth of the tumors is shown as A. (**C**) Western blot analysis showed the expression level of Aurora-A in tumor samples from B. (**D**) Immunohistochemistry analysis showed the expression of Annexin V in tumor tissues obtained from B.

To investigate the effect of overexpressed *miR-137* in tumor progression, *miR-137* stable expression cells were used to perform *in vivo* xenograft animal experiments. The results showed that tumor growth is halted in IPTG-induced *miR-137* stable cells but not the vector control cells (Figure 6A). The growth of xenograft tumors in mice supplied with IPTG was largely slower than in mice supplied with normal drinking water (Figure 6B). The expression of *miR-137* and *Aurora-A* mRNA in tumors collected from IPTG-treated or untreated mice was determined by RT-qPCR (Supplementary Figure S10A and S10B); the protein expression level of Aurora-A was decreased in tumor tissues from mice supplied with IPTG water (Figure 6C and Supplementary Figure S10C). Immunohistochemistry further showed that Annexin V was increased in tumors from IPTG-treated mice (Figure 6D and Supplementary Figure S10D). These results suggest that enforcing the expression of *miR-137* can effectively inhibit tumor growth that may result from the inhibition of target gene expression and induction of apoptosis.

### Loss of *miR-137* expression can predict the predisposition of colorectal carcinogenesis

**Figure 7:**
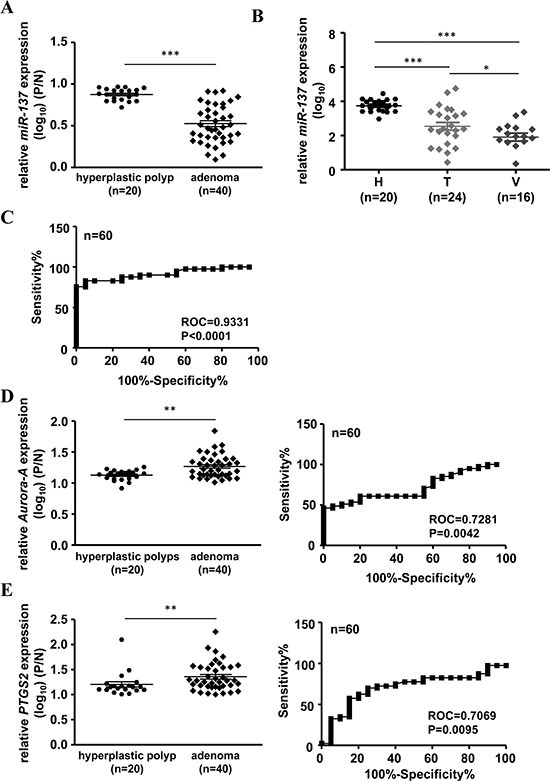
Loss of *miR-137* expression may serve as a potential biomarker in colon polyps to predict the predisposition of colorectal carcinogenesis and the potential to be a target for cancer therapy or prevention (**A**) The expression level of *miR-137* was determined in human colon polyps diagnosed as hyperplastic (*n* = 20) or adenoma (*n* = 40). (**B**) Expression levels of *miR-137* in hyperplastic (H), tubular (T) and villous (V) polyps. (**C**) ROC curve analysis of *miR-137* expression level from (A). (**D**–**E**) Expression levels and ROC analysis of *Aurora-A* (D) or *PTGS2* (E) mRNA in human colon polyps. *P < 0.05, **P < 0.01, ***P < 0.001 by Mann-Whitney *U-test*.

Our previous results showed that the expression level of *miR-137* is decreased not only in colorectal cancer tissues but also in colon polyps (Table 1, Figure 1 and Supplementary Figure S1C). It is well recognized that adenomatous polyps have a higher risk than hyperplastic polyps to develop colorectal cancer. Therefore, we checked the correlation with the expression level of *miR-137* in adenomatous polyps and hyperplastic polyps. In total, 40 adenomatous polyps (include tubular and villous tubular adenomas) and 20 hyperplastic polyps were collected to analyze the association with the expression level of *miR-137*. The results showed that *miR-137* expression was lower in adenoma but not in the hyperplastic polyps (Table 2 and Figure 7A). When checking the expression level in different types of polyps, we found that *miR-137* was more decreased in villous polyps, which have a higher risk for CRC development, than in tubular polyps (Table 3 and Figure 7B). Interestingly, the methylation percentage of *miR-137* was increased from normal mucosa, tubular polyps to villous polyps (Supplementary Figure S11). ROC (receiver operating characteristic curve) analysis suggested that the loss of *miR-137* expression in adenomatous polyps shows excellent discrimination for colorectal cancer formation (Figure 7C). Additionally, the expression level and ROC analysis of *Aurora-A* mRNA and *PTGS2* mRNA in adenomatous polyps and hyperplastic polyps showed a comparable result to that of *miR-137* (Figure 7D–7E). These results imply that *miR-137*, *Aurora-A* mRNA and *PTGS2* mRNA have the potential to act as a biomarker to predict colorectal cancer development.

**Table 2:** Association between *miR-137*, *Aurora-A* and *PTGS2* expression with colon hyperplastic polyps and adenoma polyps

	Hyperplastic polyp (*n* = 20)	Adenoma (*n* = 40)	*p*-value
**Age**			0.755
30–50 y/o (*n* = 9)	3 (5.0%)	6 (10.0%)	
51–80 y/o (*n* = 51)	17 (28.3%)	34 (56.7%)	
**Gender**			0.206
Male (*n* = 45)	13 (21.7%)	32 (53.3%)	
Female (*n* = 15)	7 (11.7%)	8 (13.3%)	
***miR-137*** **(mean log_10_ = 2.79)**			**< 0.0001**
high (log_10_ > 2.79)	20 (33.3%)	12 (20.0%)	
low (log_10_ < 2.79)	0 (0%)	28 (46.7%)	
***Aurora-A*** **(mean log_10_ = 4.64)**			**< 0.0001**
high (log_10_ > 4.64)	1 (1.7%)	23 (38.3%)	
low (log_10_ < 4.64)	19 (31.7%)	17 (28.3%)	
***PTGS2*** **(mean log_10_ = 5.26)**			**< 0.0001**
high (log_10_ > 5.26)	1 (1.7%)	26 (43.3%)	
low (log_10_ < 5.26)	19 (31.7%)	14 (23.3%)	

Bold values indicate statistically significant (Chi square).

**Table 3:** Association between *miR-137*, *Aurora-A* and *PTGS2* expression with different types of colon polyp

	Hyperplastic polyp (*n* = 20)	Tubular (*n* = 24)	Villous (*n* = 16)	*p*-value
**Age**				0.461
30–50 y/o (*n* = 9)	3 (5.0%)	4 (6.7%)	2 (3.4%)	
51–80 y/o (*n* = 51)	17 (28.3%)	20 (33.3%)	14 (23.3%)	
**Gender**				0.444
Male (*n* = 45)	13 (21.7%)	19 (31.7%)	13 (21.7%)	
Female (*n* = 15)	7 (11.7%)	5 (8.3%)	3 (5.0%)	
***miR-137*** **(mean log_10_ = 2.79)**				**< 0.0001**
high (log_10_ > 2.79)	20 (33.3%)	10 (16.7%)	2 (3.3%)	
low (log_10_ < 2.79)	0 (0%)	14 (23.3%)	14 (23.3%)	
***Aurora-A*** **(mean log_10_ = 4.64)**				**< 0.0001**
high (log_10_ > 4.64)	1 (1.7%)	11 (18.3%)	12 (20.0%)	
low (log_10_ < 4.64)	19 (31.7%)	13 (21.7%)	4 (6.7%)	
***PTGS2*** **(mean log_10_ = 5.26)**				**< 0.0001**
high (log_10_ > 5.26)	1 (1.7%)	12 (20.0%)	14 (23.3%)	
low (log_10_ < 5.26)	19 (31.7%)	12 (20.0%)	2 (3.3%)	

Bold values indicate statistically significant (Chi square).

## DISCUSSION

Downregulation of *miR-137* was found in many cancers, such as melanoma, head and neck carcinoma, breast cancer, gastric cancer and CRC, by inhibiting cancer cell proliferation, metastasis and invasion [16, 26–29]. In 2010, Balaguer F *et al.* reported that the epigenetic silencing of *miR-137* is an early event in colorectal adenoma [13]. It is well recognized that the adenoma-carcinoma sequence is the standard process for colorectal cancer formation from normal mucosa, to adenoma to carcinoma. The formation of colon polyps is the first stage of pre-cancerous lesions of CRC. Our results showed that *miR-137* is down-regulated in colon polyps, supporting the conclusion of the early silencing of *miR-137* in colorectal adenoma. According to the histological studies, colorectal polyps can be simply classified into two groups–non-neoplastic and neoplastic polyps–according to their malignant potency [30]. Importantly, the expression level of *miR-137* is gradually decreased in hyperplastic, tubular and villous polyps (Figure 7B and Table 3). Furthermore, the results from ROC analysis showed that the epigenetic silencing of *miR-137* not only occurs in the early stage of the neoplastic pathway but also serves as a biomarker to predict the tendency toward to CRC formation (Figure 7C).

In this study, we found that *Aurora-A* is overexpressed not only in the tumorous tissues of CRC but also in the colon polyps (Figure 4B). This is the first report to demonstrate the overexpression of Aurora-A in tissues with pre-cancerous lesions. Our results indicated that the expression of *Aurora-A* mRNA and *miR-137* is inversely correlated in human colorectal cancer tissues and colon polyps (Figure 4C). Although it is reasonable to detect the overexpressed Aurora-A in colon neoplastic tissue polyps, the clinical significance of overexpressed Aurora-A in the early stage of colorectal adenoma remains unclear and needs to be further investigated. Indeed, ROC analysis supports the potential role of Aurora-A in predicting the tendency of CRC development (Figure 7D). In addition to *Aurora-A*, other potential targets of *miR-137* were also characterized, including *PTGS2*, which also plays an important role in colorectal carcinogenesis [21]. By Q-PCR and ROC analysis, we found that the expression of *PTGS2* is negatively correlated with *miR-137* in human colorectal cancer tissues and polyps, as well as acts as a biomarker that can predict the tendency toward to CRC formation (Figures 4F and 7E).

It was previously indicated that the overexpression of *miR-137* can induce cell cycle G1 arrest in gastric cancer cells through targeting *Cdc42* [15]. However, in our study, the enforced expression of *miR-137* in CRC cell lines induces obvious G2/M arrest and an increase in phosphor-histone H3/Serine 10, both of which result from the depressed expression of *Aurora-A* (Figure 5A, 5B, and Supplementary Figure S8). Importantly, the overexpression of Aurora-A in *miR-137*-expressing cells can reverse those effects (Figure 5D and 5E). The difference between the previous study [15] and this study may be due to the different stages of cancer development. The silenced expression of *miR-137* in the early stage of carcinogenesis may contribute to the neoplastic growth through the action of *Aurora-A*, whereas in the late stage of carcinogenesis, the loss of *miR-137* expression can promote cancer cell metastasis by the increased expression of *CDC42*. Using the xenograft animal model, we demonstrated that the enforced expression of *miR-137* can inhibit tumor growth, and the expression of *Aurora-A* in *miR-137*-expressing tumor tissues is repressed (Figure 6 and Supplementary Figure S10). These results strongly suggest that the loss of *miR-137* expression may promote the neoplastic processing through the impaired ability in *Aurora-A* inhibition and also imply that *miR-137* has the potential to be a therapeutic miRNA.

In conclusion, our study indicates that the epigenetic silencing of *miR-137* can occur as early as during neoplastic growth, and the loss of *miR-137* expression can act a biomarker to predict the predisposition of CRC formation. The expression of *miR-137* gradually decreases during the process of colorectal carcinogenesis. Our result provides the clinical significance of *miR-137* in the early stage of colorectal cancer development by directly inhibiting *Aurora-A* and *PTGS2* expression. Furthermore, we propose the potential to consider *miR-137* as a therapeutic miRNA in cancer therapy.

## MATERIALS AND METHODS

### Samples of clinical specimens

Studies of clinical specimens were conducted according to a laboratory protocol approved by the Institutional Review Board of National Cheng Kung University Hospital (B-ER-103-228), and were in accordance with the *Helsinki Declaration* of 1975, as revised in 1983.

### Cell culture

Human colon cancer cells, SW480 cells and HCT116 cells were grown at 37°C under 5% CO_2_ in 10-cm plastic dishes containing 10 ml of Leibovitz's L-15 medium and RPMI medium 1640, respectively, supplement with 10% fetal bovine serum, 100 μg/ml streptomycin, and 100 U/ml penicillin. The precursor *miR-137* was cloned into an IPTG-inducible pLAS1w.3xLacO expression vector (a kindly gift from Dr. Ju-Ming Wang). The stable clones were established in HCT116 cells and selected by 1.5 μg/ml of puromycin. The cells were treated with 62.5 μM of IPTG to induce the expression of *miR-137*.

### Total RNA purification and real-time PCR

The total RNA was extracted using TRIzol reagent (Invitrogen, Carlsbad, CA) according to the manufacturer's instructions. To detect mRNA expression, reverse transcription was performed with 1 μg of total RNA using MultiScribe™ MuLV Reverse Transcriptase (Applied Biosystems, Forster, CA). Real-time PCR was performed using the SYBR Advantage qPCR premix (Bio-Rad, Hercules, CA) in a CFX96™ Real-Time System and C1000 ™ Thermal Cycler (Bio-Rad), and the reactions were performed using the following conditions for 40 cycles: 95°C for 15 sec, 60°C for 10 sec, and 72°C for 5 sec. The primer sequences were as follows: forward and reverse primers for human *Aurora-A* mRNA: 5′-AATGCCCTGTCTTACTGTCATTC-3′ and 5′-TCCAGAGATCCACCTTCTCATC-3′; for human *PTGS2* mRNA: 5′-CCCTTCTGCCTGACACCTTT-3′ and 5′-TTCTGTACTGCGGGTGGAAC-3′; for human *CDK6* mRNA: 5′-TCACACCGAGTAGTGCATCG-3′ and 5′-CAAGACTTCGGGTGCTCTGT-3′; and for human *CDC42* mRNA: 5′-AGGCTGTCAAGTATGT GGAGTG-3′ and 5′-GCTCTTCTTCGGTTCTGGAGG-3′. The data were analyzed using Bio-Rad CFX Manager software version 1.5. The relative amount of the target gene was normalized to that of *actin* of the same cDNA. To detect the expression of *miR-137*, the cDNA was synthesized using the MicroRNA reverse transcription kit (TaqMan; Applied Biosystems), and the *miR-137* and endogenous control *U6* small nuclear RNA (snRNA) expression levels were analyzed using the TaqMan microRNA Assay kit (Applied Biosystems) according to the manufacturer's instructions. Briefly, 900 ng of total RNA was combined with 6 μl of the RT primer pool (the final concentration was 0.05×), 1.5 μl of 10× RT buffer, 0.3 μl of 100 mM dNTP mix, 3 μl of 50 U/μl MultiScribe Reverse Transcriptase, and 0.19 μl of 20 U/μl RNase inhibitor in a total volume of 15 μl. Each sample was run in individual 0.2 ml-tubes using the following parameters: 16°C for 30 min, 42°C for 30 min, and 85°C for 5 min, followed by holding at 4°C. TaqMan Real-time PCR was performed using 2× TaqMan Universal PCR Master Mix II, and No AmpErase UNG (Applied Biosystems) in a CFX96™ Real-Time System and C1000 ™ Thermal Cycler (Bio-Rad), and the reactions were performed using the following conditions for 40 cycles: 95°C for 15 sec, and 60°C for 1 min. The TaqMan microRNA assay analyzed *hsa-miR-137* (Assay ID 001129) and endogenous control *U6* small nuclear RNA (Assay ID 001973). The expression of *miR-137* was normalized to that of *U6* snRNA.

### 3′-UTR luciferase reporter assay

The *Aurora-A* and *PTGS2* 3′-UTR luciferase constructs were purchased from OriGene Technologies (Rockville, MD). The cells were transfected with 250 ng of pMirTarget-*Aurora-A*-3′-UTR or pMirTarget-*PTGS2*-3′-UTR luciferase construct and 750 ng pMiR-137 or control vector using Lipofectamine 2000 (Invitrogen). Three nucleotides within the *miR-137* seed sequence in the pMirTarget-*Aurora-A*-3′-UTR construct were mutated. After 48 hours, the cells were harvested and assayed using the Dual-Luciferase Reporter Assay System (Promega, Madison, WI). A *Renilla* luciferase construct was used as a normalizing control for all luciferase assays. Experiments were conducted in triplicate.

### Methylation-specific polymerase chain reaction (MSP)

Genomic DNA from human specimens was isolated using an automated DNA extraction system (QuickGene, Neyagawa-shi, Osaka, JP) according to the manufacturer's instructions, and then the unmethylated cytosine was converted into uracil using the EpiTect Bisulfite Kit (Qiagen, Hilden, Germany). The primer sequences used to analyze *miR-137* promoter methylation were designed using Methyl Primer Express Software v1.0 (Applied Biosystems) and were as follows: methylated alleles: 5′-GTAGCGGTAGCGGTAGTAGC-3′ and 5′- ACCG CTAATACTCTCCTCGA-3′; unmethylated alleles: 5′- GTAGTAGTGGTAGTGGTAGTAGT-3′ and 5′-CCTAC CACTAATACTCTCCTCAA-3′. MSP reaction was performed using the following condition for 35 cycles: 95°C for 30 sec, 52°C for 30 sec, and 72°C for 40 sec. The MSP results were evaluated using 2% agarose gel electrophoresis.

### Quantitative bisulfite pyrosequencing

DNA fragments were bisulfite converted and amplified with a primer set (Supplementary Figure S2A). The primers for PCR amplification and pyrosequencing were designed using PyroMark Assay Design 2.0 (Qiagen). The sequences of the PCR primers were as follows: forward primer: 5′-GAGAGGTTATTTGGATTTGGGTAGGAA-3′; reverse primer: 5′-CACCCAAAAAAATCAAAAAACCAAACT AC-3′; pyrosequencing primer: 5′-GGGTTTAGAGAG TAGTAAGA-3′. To determine the methylation level of *miR-137*, 7 CpG sites on *miR-137* were used for pyrosequencing analysis (Supplementary Figure S3). Quantitative DNA methylation analysis of pyrosequencing was carried out using a PyroMark Q24 System (Qiagen) and was analyzed using PyroQ-CpG 1.0.9.

### Xenograft animal studies

Animal experiments were performed according to the Institutional Animal Care and Use Committee (IACUC) at the laboratory animal center of National Cheng Kung University. HCT116 stable clones with control vector or inducible *miR-137* were subcutaneously injected (1 × 10^6^) into the left or right flank of 5- to 6-week old female NOD-SCID mice (*n* = 6). On the following day, the mice were fed 10 mM IPTG in the drinking water to induce miR-137 expression. Tumor growth was measured using a caliper twice per week. Additionally, in another experiment, HCT116 stable clones with inducible miR-137 were subcutaneously injected (1 × 10^6^) into the bilateral flank of 5- to 6-week old female NOD-SCID mice (*n* = 10). After the tumors reached approximately 100 mm^3^, the mice were separated into two groups randomly and fed regular water or 10 mM IPTG in the drinking water to induce *miR-137* expression. The tumor volume was calculated using the following equation: (length × width^2^)/2. To determine the presence of apoptotic cells in the subcutaneous tumors, paraffin-embedded sections was used to perform the immunohistochemical staining using anti-Annexin V antibodies (AP6580b, ABGENT, San Diego, CA).

### Immunoblotting analysis

Total protein isolated from cultured cell lines or tumor tissues from xenograft animal studies were lysed in modified RIPA buffer (50 mM Tris-HCl at pH 7.4, 150 mM NaCl, 1 mM EDTA, 1% NP-40, 0.25% sodium deoxycholate) with 1 mM DTT, 10 mM NaF, 1 mM PMSF, 1 μg/ml of aprotinin, 1 μg/ml of leupeptin, 1 mM Na_3_VO_4_, and the phosphatase inhibitor cocktail to be analyzed. Following lysis, the lysates were resolved on an SDS-containing 10% polyacrylamide gel were transferred to a polyvinylidene difluoride nylon (PVDF) membrane and were probed with specific antibodies. The specific bands were detected by horseradish peroxidase-conjugated antibody and were revealed by Western Lighting^®^ Plus-ECL (PerkinElmer, Waltham, MA) and X-ray film (Fujifilm, Tokyo, JP). The antibodies used were PARP-1 (F2), phospho-histone H3 and GAPDH from Santa Cruz Biotechnology (Santa Cruz, CA), Aurora-A [35C1] from GeneTex (Irvine, CA), and caspase-3 from IMGENEX (San Diego, CA).

### Immunochemical staining

Formalin-fixed paraffin-embedded sections of *xenograft* tumor tissues were mounted on glass slides, deparaffinized with xylene and followed to rehydrate by a graded ethanol series. After microwaving in citrate-phosphate buffer (pH 6.0) to retrieve the antigen, the slides were incubated with 3% H_2_O_2_ at room temperature to block endogenous peroxidase activity. The sections were incubated with primary antibody at room temperature. Detection was followed with streptavidin-biotinylated peroxidase-conjugated reagents (LSAB^+^ kit: Dako, Carpentaria, CA) and 3,3′ diaminobenzidine (DAB) as the chromogen, and the tissues were counterstained with hematoxylin.

### Immunofluorescence assay

The cells were seeded on sterile glass coverslips for 18–24 hours. After treatment, the coverslips were removed from the medium and were washed once with PBS and then were fixed in 3.7% formaldehyde for 10 minutes at room temperature. The cells were rinsed in PBS and were permeabilized in 0.1% Tween-20/PBS for 10 minutes. The cells were then incubated with the primary antibodies diluted in 3% BSA/PBS for 1 hour at 37°C, were washed in 0.1% Tween-20/PBS and were labeled for 1 hour at 37°C with appropriate Alexa 568-conjugated secondary antibodies. The cells were mounted in ProLong^®^ Gold antifade reagent with DAPI (Invitrogen), and images were obtained with an immunofluorescence microscope.

## SUPPLEMENTARY MATERIALS FIGURES AND TABLES




