# Supplementary material for: Epigenetic silencing of miR-137 contributes to early colorectal carcinogenesis by impaired Aurora-A inhibition
Source: Oncotarget. 2016 Oct 18;7(47):76852–66. doi: 10.18632/oncotarget.12719 (PMC5363554; doi:10.18632/oncotarget.12719)
Supplement: Supplementary file 1 [file oncotarget-07-76852-s001.pdf]

Epigenetic silencing of *miR-137* contributes to early colorectal carcinogenesis by impaired *Aurora-A* inhibition

Supplementary Materials

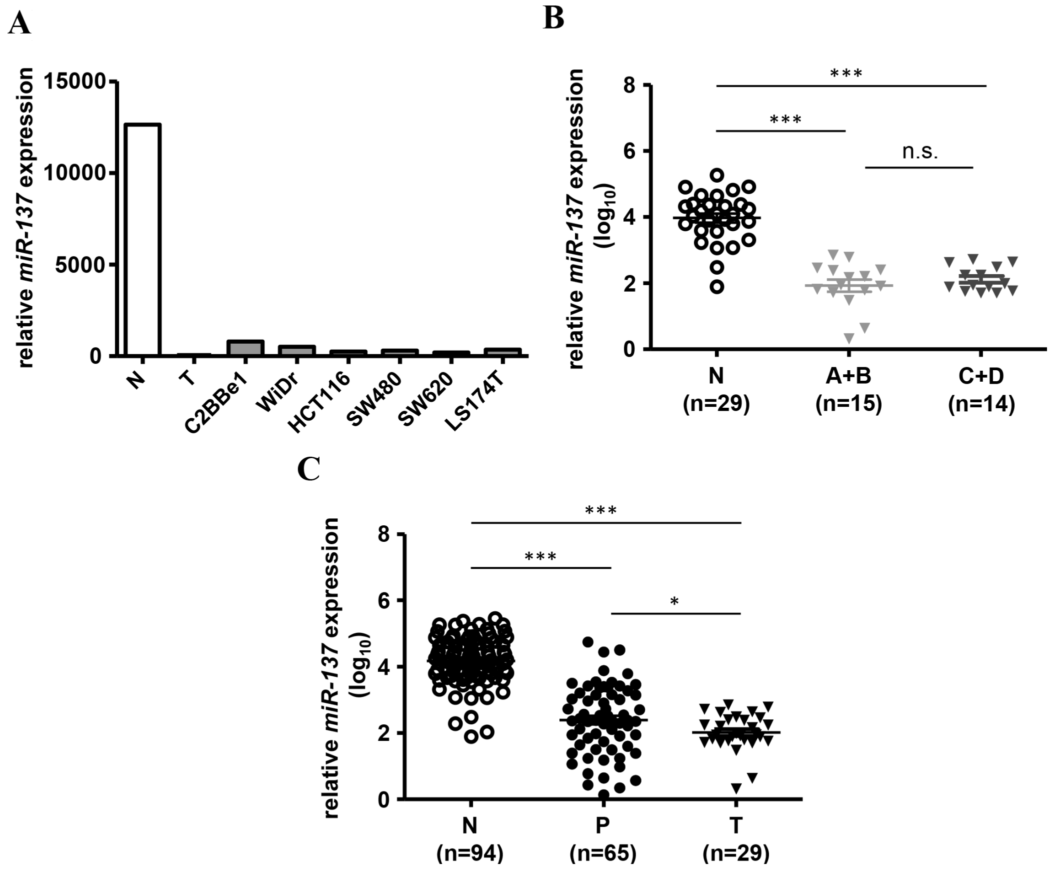

Supplementary Figure S1: Expression of *miR-137* in colorectal cancer cell lines, colorectal cancer tissues and colon polyps. (**A**) TaqMan Q-PCR analysis of *miR-137* expression in the six CRC cell lines C2BBe1, WiDr, HCT116, SW480, SW620, and LS174T. A pair of human colorectal cancer tissue (T) and adjacent normal tissue (N) from figure 1A was used as control. (**B**) Comparison of the *miR-137* expression level in CRC tissues with stage A + B and stage C + D disease. These specimens are the same as those in Figure 1A. (**C**) Comparison of the *miR-137* expression level in normal (N), colon polyps (P) and CRC tissues (T). The specimens are the same as those in Figure 1A and 1B.

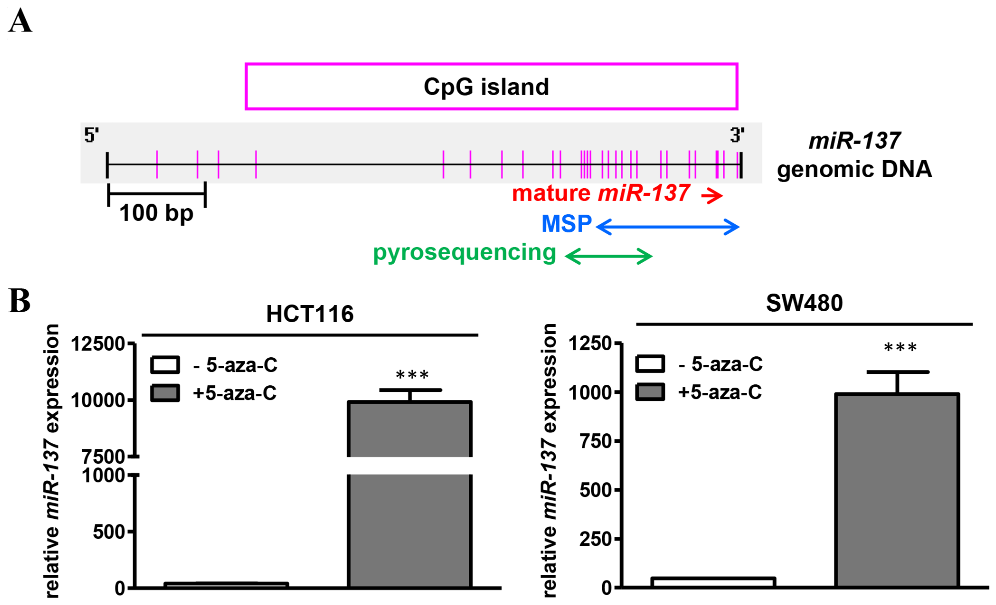

Supplementary Figure S2: Epigenetic regulation of *miR-137* in colorectal cancer cells. (**A**) The schematic illustration of the *miR-137* promoter shows the region of CpG islands. The regions of mature *miR-137* and to detect the promoter methylation by methylation-specific PCR (MSP) or pyrosequencing analysis are shown. (**B**) HCT116 and SW480 cells were treated with (+) or without (–) 2.5 µM 5-aza-C for 7 days and then were harvested to analyze the expression of *miR-137* by TaqMan Q-PCR.

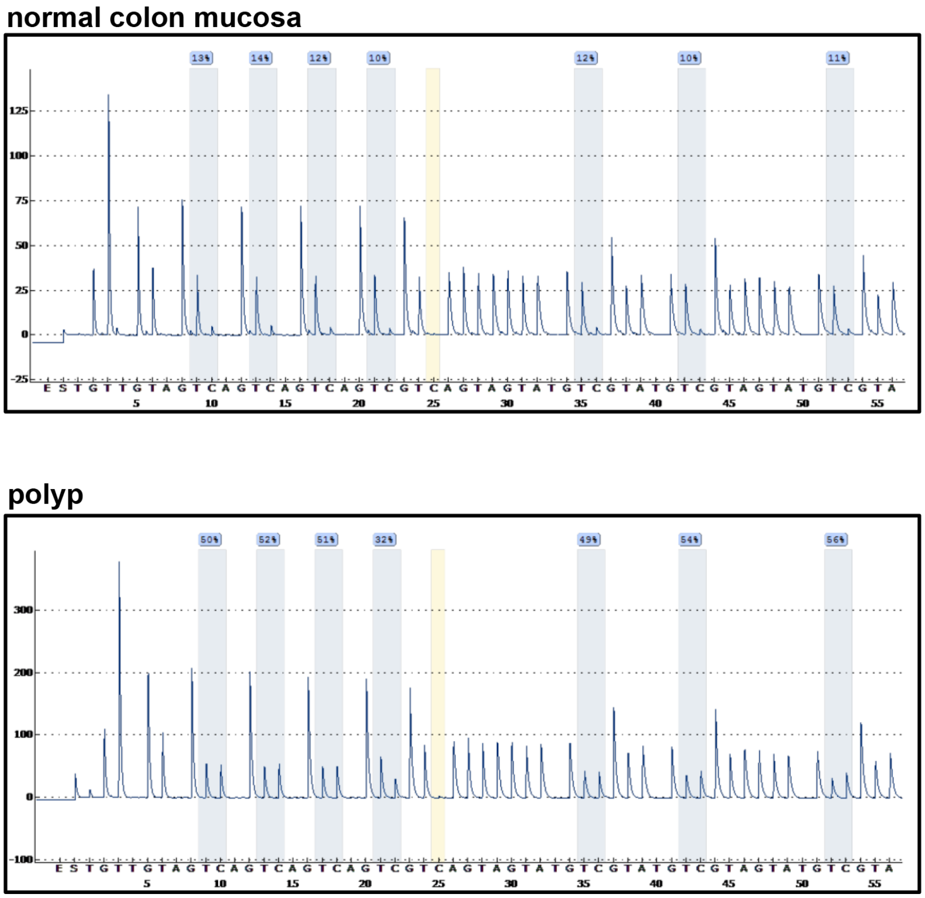

Supplementary Figure S3: Pyrosequencing analysis of *miR-137* in colorectal polyps and their adjacent non-tumorous tissues. Seven CpG sites within *miR-137* were analyzed, and one representative result of a paired specimen is shown. The methylation percentage of normal colon mucosa ranged from 11% to 14% (upper), whereas that of polyps ranged from approximately 32% to 56% in the representative paired sample (lower).

A

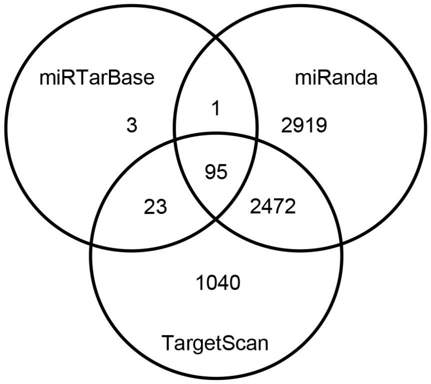

B

Category	Gene Symbol
Disease (by Biomarkers)	
Carcinoma, Ductal	Akt2, Aurora-A, c-Kit, CDC42, CDK6, COX2(PTGS2), CtBP1, ERR1, GCR, H3F3B, JAG1, KLF4, MTDH, NCOA2, NCOA3, TEF, TGFβ2, YBX1
Neoplasms, Ductal, Lobular, and Medullary	Akt2, Aurora-A, c-Kit, CDC42, CDK6, COX2(PTGS2), CtBP1, ERR1, GCR, H3F3B, JAG1, KLF4, MTDH, NCOA2, NCOA3, TEF, TGFβ2, YBX1
Carcinoma, Ductal, Breast	Akt2, Aurora-A, c-Kit, CDC42, CDK6, COX2(PTGS2), CtBP1, ERR1, GCR, JAG1, KLF4, MTDH, NCOA2, NCOA3, TGFβ2, YBX1
GO Processes	
Postive regulation of gene expression	Akt2, Bcl11b, c-Kit, CDC42, CDK6, CNN2, COQ7, CtBP1, ERR1, FOXK1, GCR, GPBP1L1, JAG1, KLF4, KDM1A, MITF, MTDH, NCOA2, NCOA3, NFYB, PAIP1, PAX9, PORA, RREB1, TBX3, TEF, TGFβ2, YBX1, ZNF326
Postive regulation of cell growth	Akt2, DBN1, GCR, H3F3B, H3F3C, NCOA3, TGFβ2
Pathway Maps	
Development TGF-beta-dependent induction of EMT via SMADs	JAG1, TGFβ2
Immune response PGE2 in immune and neuroendocrine system interactions	COX2(PTGS2), GCR, PTGES2
Cell adhesion Chemokines and adhesion	Akt2, CDC42, DBN1
Process Networks	
Development EMT regulaton of Epithelial-to-mesenchymal transition	Akt2, CDC42,COX-2(PTGS2), JAG1, TGFβ2
Cell cycle G1-S growth factor regulation	Akt2, CDK6, TGFβ2
Cell cycle G0-G1	CDK6, CtBP1, E2F6

C

```
Aurora-A 3′-UTR (374-380)   5′ ...GUUAAGGAGCUGUGCAAUAA...
PTGS2 3′-UTR (911-917)      5′ ...ACCUUUAAAAUAAGCAAUAA...
PTGS2 3′-UTR (1675-1681)    5′ ...UAAGUAGUUCUCAGCAAUAA...
CDK6  3′-UTR (7133-7139)    5′ ...UAUGUAUCAGCUAGCAAUAU...
CDK6  3′-UTR (4214-4220)    5′ ...GCAACCACAUGAAGCAAUAC...
CDC42 3′-UTR (98-104)       5′ ...AAAAUUCGUUUUUGCAAUAA...
hsa-miR-137                 3′  AUGCGCAUAAGAAUUCGUUAUU
```

Supplementary Figure S4: Prediction of *miR-137* potential target genes. (**A**) The potential target genes of *miR-137* were searched by two free software programs, miRanda and TargetScan, and confirmed by miRTarBase. (**B**) MetaCore analysis of *miR-137* potential target genes. (**C**) Four genes, *Aurora-A*, *PTGS2*, *CDK6* and *CDC42*, were chosen to test the accuracy of prediction. The potential targeting sites of *miR-137* in the 3′-UTR of *Aurora-A*, *PTGS2*, *CDK6*, and *CDC42* are shown.

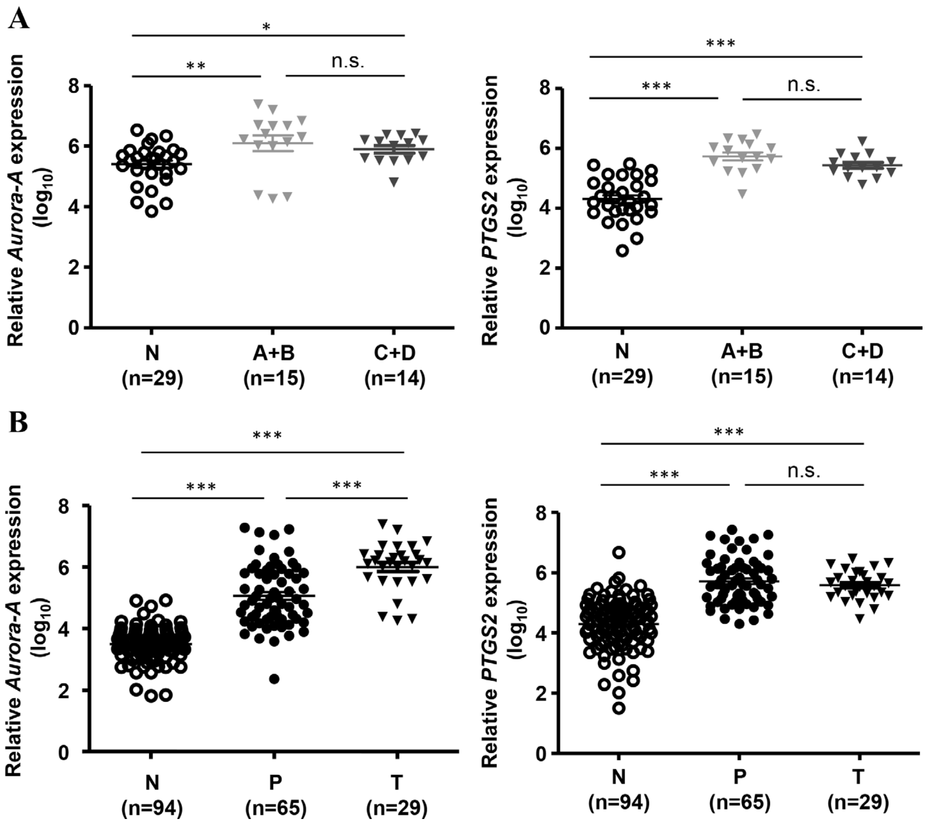

Supplementary Figure S5: The expression level of *Aurora-A* mRNA and *PTGS2* mRNA in human colon mucosa, colon polyps and colorectal cancer tissues. (**A**) Comparison of the *Aurora-A* (left) and *PTGS2* (right) mRNA expression levels in CRC tissues with stage A + B and stage C + D disease. These specimens are the same as those in Figure 1A. (**B**) Comparison of the *Aurora-A* (left) and *PTGS2* (right) mRNA expression levels in normal mucosa (N), colon polyps (P) and CRC tissues (T). Specimens are the same as Figure 1A and 1B.

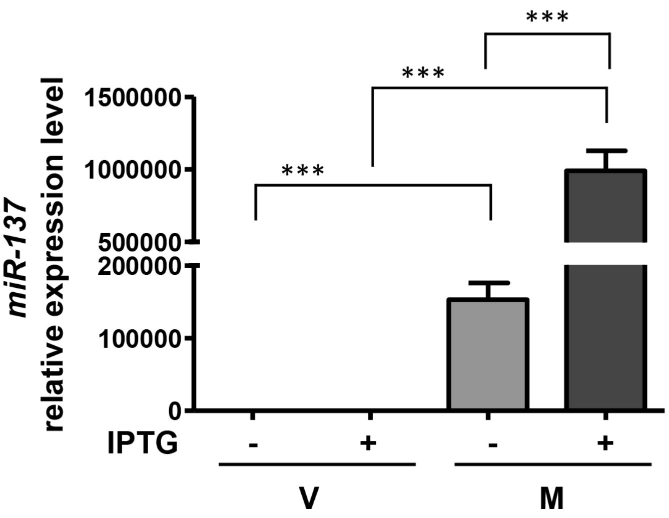

Supplementary Figure S6: The inducible *miR-137* stable cell line can effectively inhibit the expression of Aurora-A mRNA. Vector control (V) or *miR-137* (M) stably expressed HCT116 cells were treated with (+) or without (–) IPTG for 48 h to induce the expression of *miR-137*.

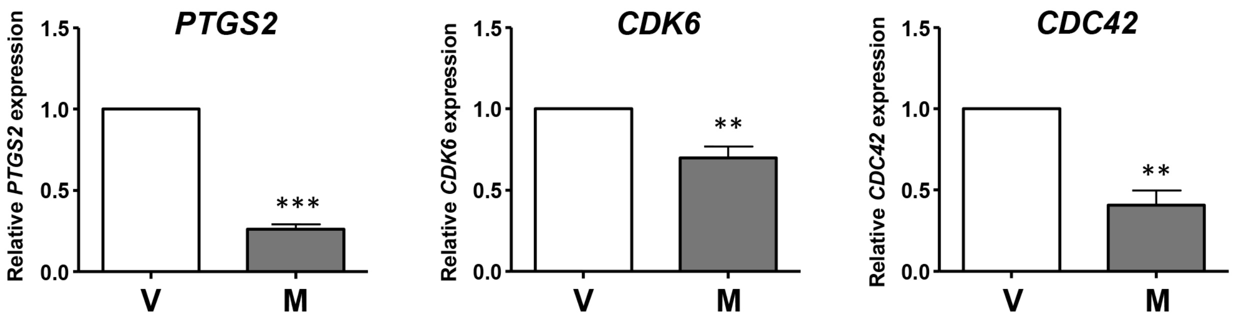

Supplementary Figure S7: Inhibitory expression of *PTGS2*, *CDK6* and *CDC42* in *miR-137* stable cell lines. IPTG-induced expression of *miR-137* inhibited the expression of *PTGS2*, *CDK6*, and *CDC42* in *miR-137* stably expressing HCT116 cell line.

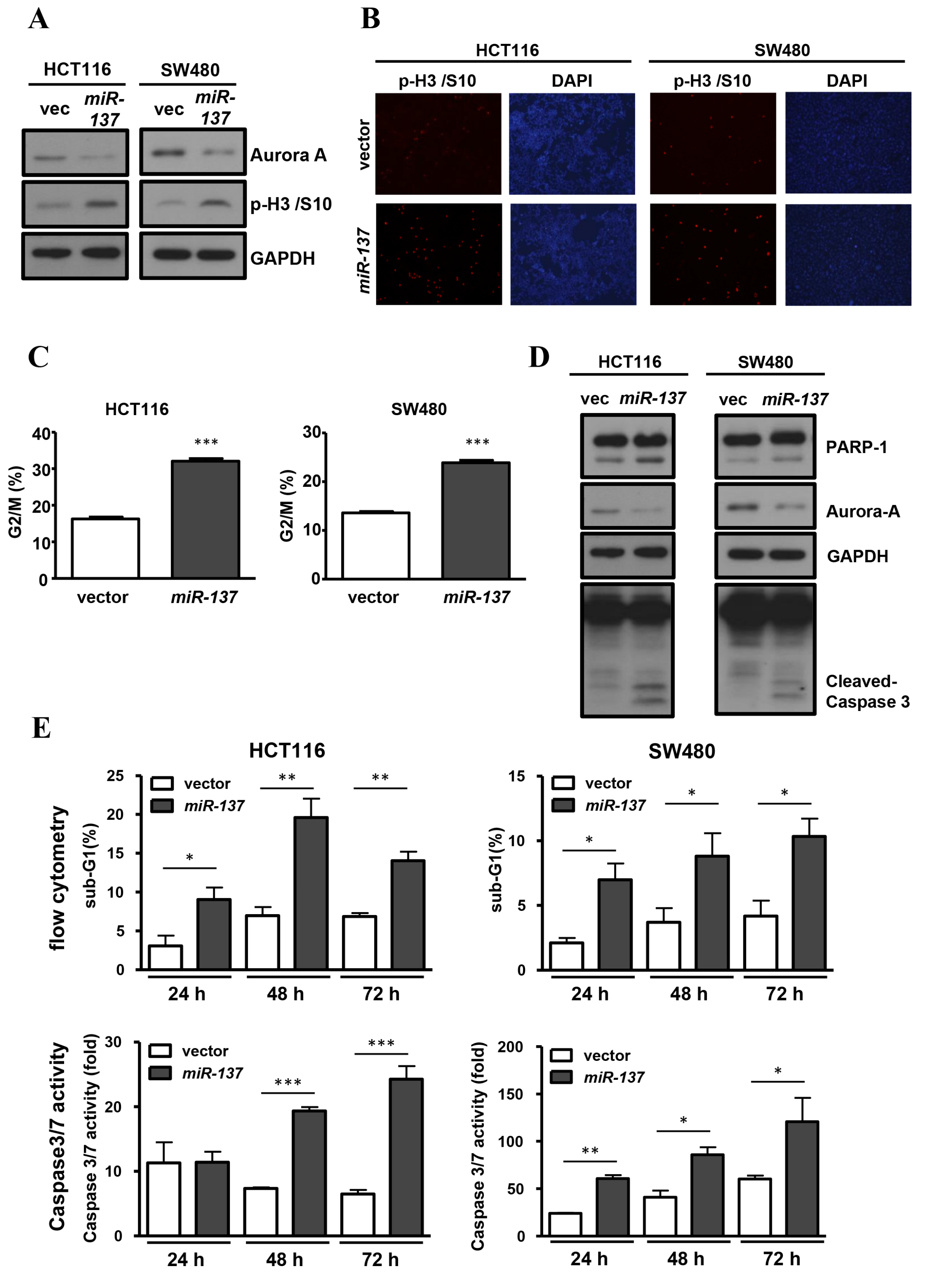

Supplementary Figure S8: The expression of *miR-137* induces G2/M arrest and apoptosis. (**A**) Western blot analysis showed the expression of phosphor-histone H3/Ser10 (p-H3/S10) in *miR-137* or vector control transiently transfected HCT116 and SW480 cells. (**B**) Immunofluorescence assay showed an increased intensity of phosphor-histone H3/Ser10 (p-H3/S10, red) in *miR-137*-overexpressing HCT116 and SW480 cells. DAPI is a DNA dye. (**C**) Flow cytometry analysis indicated that the expression of *miR-137* induces G2/M arrest in HCT116 and SW480 cells. (**D**) Western blot analysis showed the expression of PARP-1, Aurora A, and Caspase-3 in *miR-137* or vector control transiently transfected HCT116 and SW480 cells. (**E**) Flow cytometry analysis demonstrated the time-dependent increase in the sub-G1 population and Caspase-3/7 activity in HCT116 and SW480 cells transiently transfected with *miR-137* or vector control.

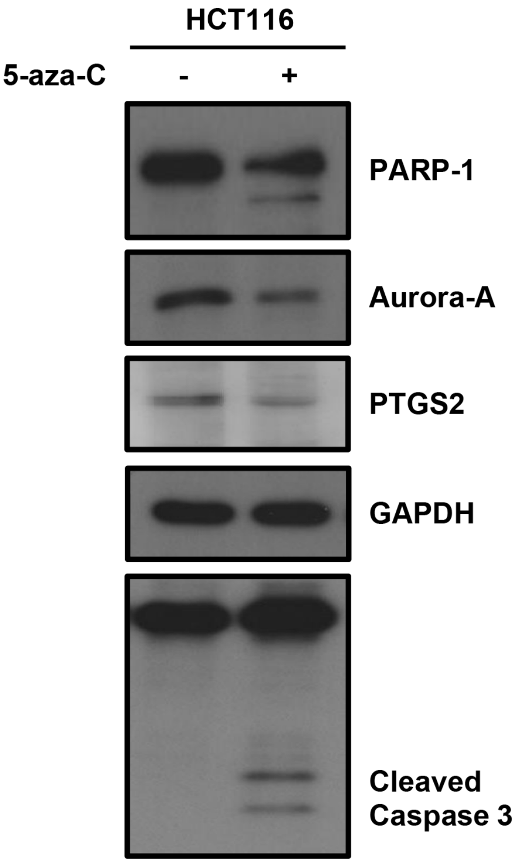

Supplementary Figure S9: The treatment of 5′-AzaC inhibits Aurora-A expression, and induces G2/M arrest and cell death. HCT116 cells were treated with (+) or without (–) 5-aza-C for 7 days and then were collected to detect the expression of PARP-1, Aurora A, PTGS2 and Caspase-3 by Western blot analysis. GAPDH was used as the loading control.

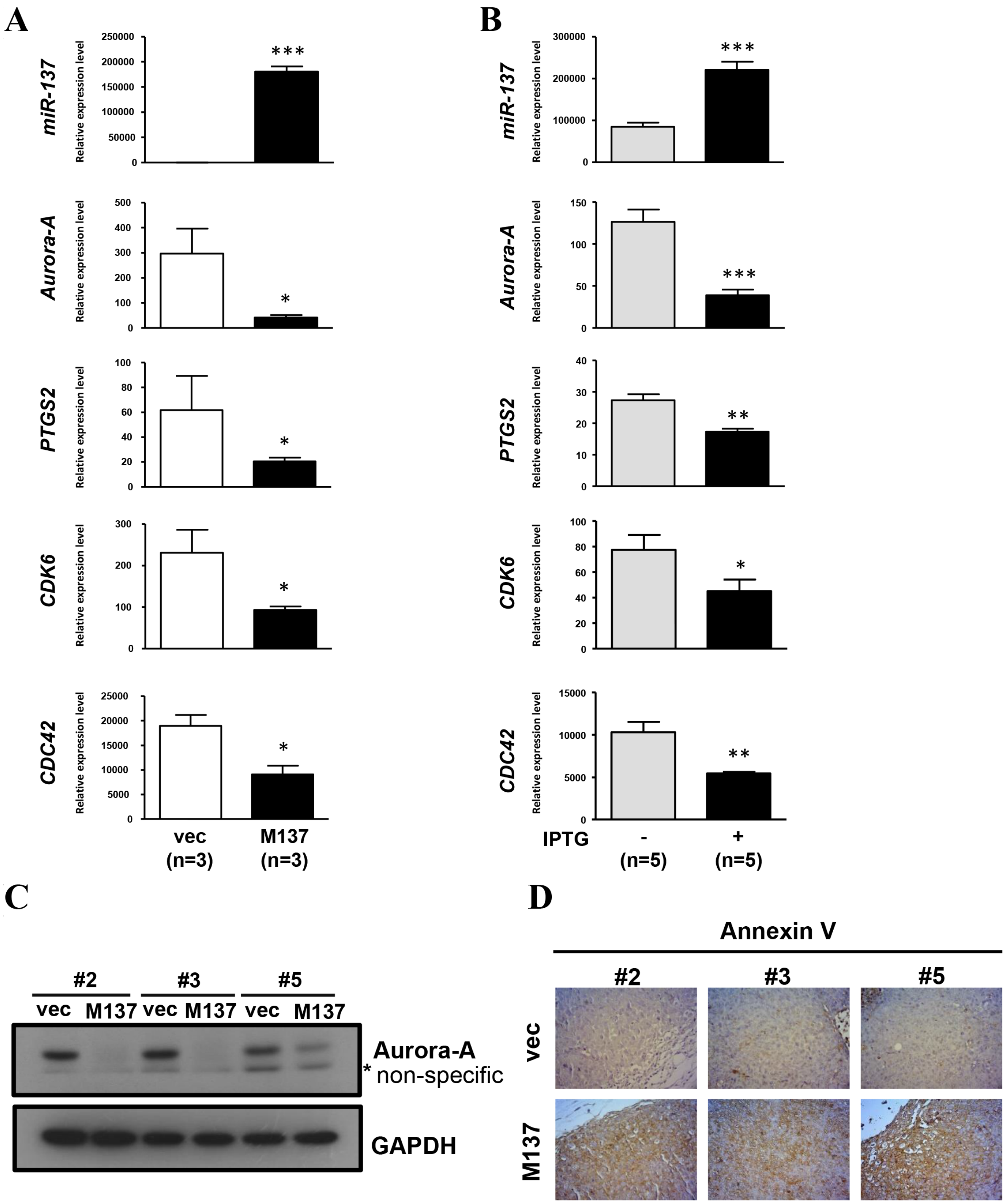

Supplementary Figure S10: Characterization of the expression level of *miR-137*, Aurora-A and Annexin V in *miR-137*-expressing *xenograft* tumor tissues. (**A**) The expression level of *miR-137* and *Aurora-A* mRNA in tumor samples from Figure 6A was determined by TaqMan Q-PCR and Q-PCR, respectively. (**B**) The expression level of *miR-137* and *Aurora-A* mRNA in tumor samples from Figure 6B was determined by TaqMan Q-PCR and Q-PCR, respectively. (**C**–**D**) Tumor samples from Figure 6A were collected to detect the expression level of Aurora-A by Western blot analysis (C) and Annexin V by immunohistochemistry assay (D).

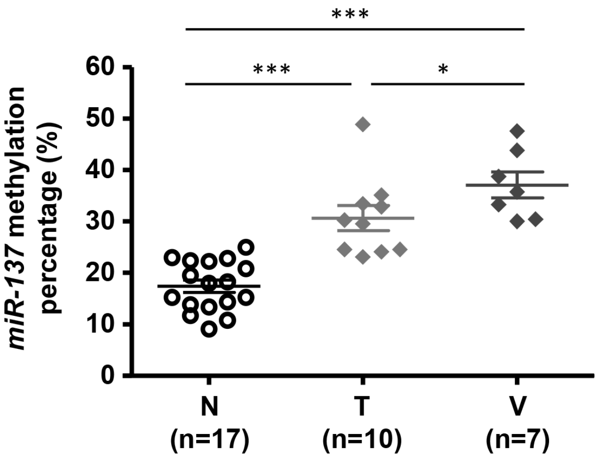

Supplementary Figure S11: Quantitative result of methylated *miR-137* in different types of human colon polyps. The quantitative results of pyrosequencing from Figure 2E were classified as normal (N), tubular (T) or villous (V) polyps to show the methylated levels of *miR-137*.

Supplementary Table S1: Raw expression level of *miR-137* in Figure 1A.

See Supplementary_Table_S1

Supplementary Table S2: Raw expression level of *miR-137* in Figure 1B.

See Supplementary_Table_S2
