# Supplementary material for: Epigenetic silencing of miR-137 contributes to early colorectal carcinogenesis by impaired Aurora-A inhibition
Source: Oncotarget. 2016 Oct 18;7(47):76852–66. doi: 10.18632/oncotarget.12719 (PMC5363554; doi:10.18632/oncotarget.12719)
Supplement: Supplementary file 2 [file oncotarget-07-76852-s002.docx]

**Supplementary Table 1. Raw expression level of *miR-137* in Figure 1A.**

**Supplementary Table 2. Raw expression level of *miR-137* in Figure 1B.**
